# Hypothyroidism, lipids, and lipidomics

**DOI:** 10.1007/s12020-023-03420-9

**Published:** 2023-06-17

**Authors:** Jacqueline Jonklaas

**Affiliations:** Division of Endocrinology, Georgetown University, Washington, DC USA

**Keywords:** Hypothyroidism, Subclinical hypothyroidism, Cholesterol, Low density lipoprotein cholesterol, Thyroid hormone, Coronary heart disease

## Abstract

**Purpose:**

Hypothyroidism is a relatively common endocrine disorder and is well documented to be associated with lipid abnormalities.

**Methods:**

A narrative review was conducted of studies describing the alterations in the lipid profile accompanying both subclinical and overt hypothyroidism.

**Results:**

Lipid abnormalities are seen with TSH values in the upper end of the accepted reference range, as well as with subclinical and overt hypothyroidism. The degree of lipid derangement is generally proportional to the degree of TSH elevation. Other factors such as age, sex, and body mass index can also influence the pattern of the lipid abnormalities seen. The most robust finding with TSH elevation is increases in the low density lipoprotein cholesterol. Thyroid hormone treatment is efficacious in reversing the lipid abnormalities in both subclinical and overt hypothyroidism.

**Conclusion:**

Given the association of lipid abnormalities with metabolic and cardiovascular disease, consideration of hypothyroidism as an important non-communicable disease may facilitate studies that test the hypothesis that thyroid hormone treatment to reverse hypothyroidism-associated lipid abnormalities may improve metabolic and cardiovascular outcomes.

## Introduction

Thyroid hormone has pervasive effects on all the organ systems of the body. This includes a profound effect on lipid metabolism. With respect to hypothyroidism, this disorder leads to both hypercholesterolemia and hypertriglyceridemia. Both overt and subclinical hypothyroidism may also be associated with insulin resistance, metabolic syndrome, and increased risk for cardiovascular disease. Analyses of the impact of overt and subclinical hypothyroidism on producing adverse changes in the lipid profile are also complicated by other factors such as body weight or body mass index, sex, and smoking. However, mounting evidence suggests that not only are overt and subclinical hypothyroidism associated with increases in cholesterol and triglycerides, but also that even increases in TSH within the normal range are associated with an unfavorable lipid profile, and metabolic and cardiovascular disease risk.

## Lipid metabolism

**Fig. 1:**
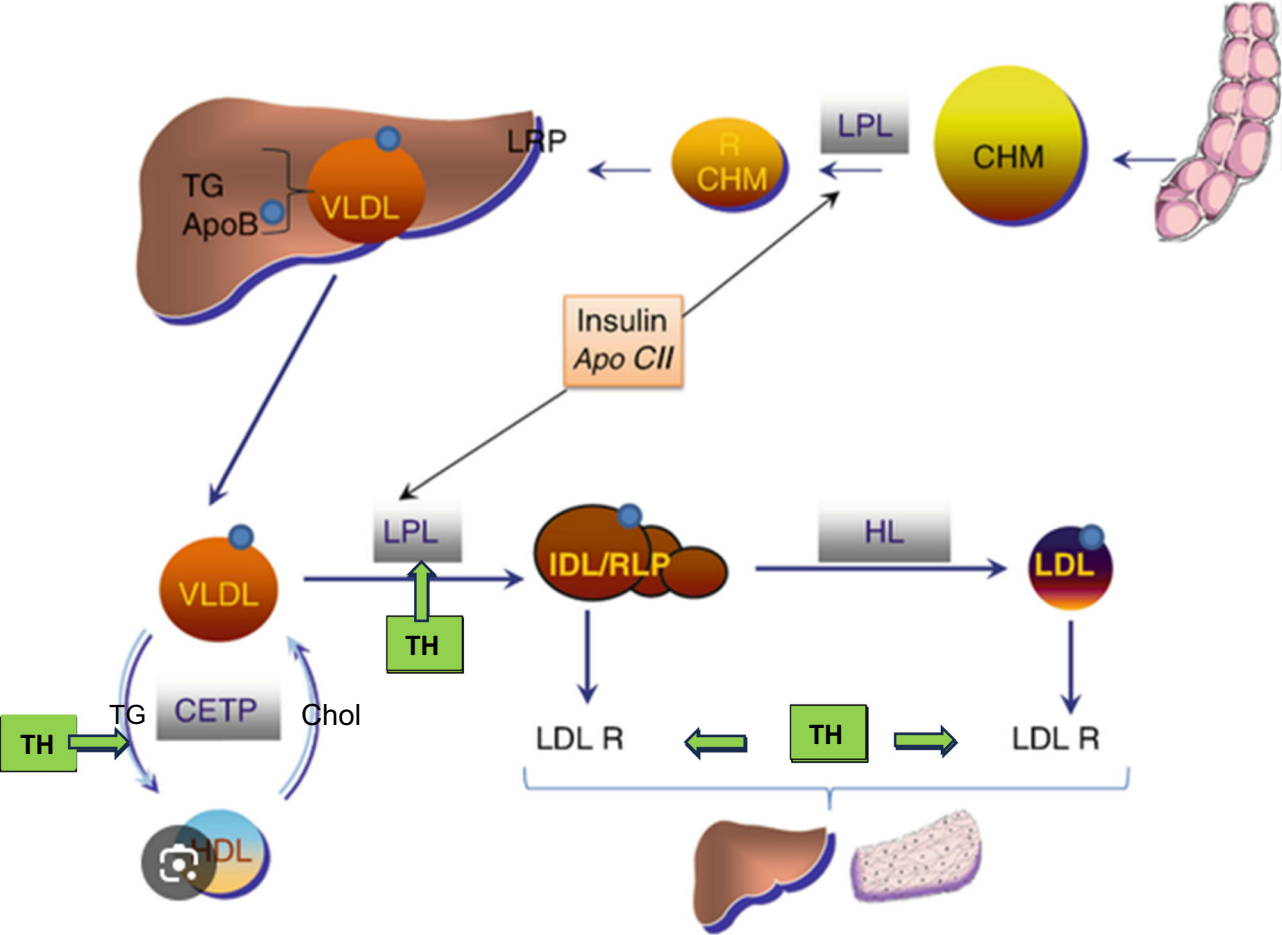
Some actions of thyroid hormone on lipid metabolism. TH = thyroid hormone. From Brenta and Schreier [45]

Cholesterol production occurs in the liver via the actions of the enzyme 3-hydroxy-3-methylglutaryl-CoA reductase. Cholesterol is then transported within the circulation by lipoproteins which are categorized according to their size and density. The lipoprotein subfraction low density lipoprotein cholesterol (LDL-C) is atherogenic, susceptible to oxidation, and is associated with the development of coronary artery disease. On the other hand, high density lipoprotein cholesterol (HDL-C) enables reverse transportation of cholesterol from the circulation into the liver and is associated with cardio-protective effects. Cholesteryl ester transfer protein (CETP) transfers cholesterol from HDL-C to LDL-C and very low density lipoprotein (VLDL), thus playing an atherogenic role. Lipoprotein lipase may play a protective role by lowering triglyceride levels through hydrolysis of triglyceride-enriched lipoproteins and facilitating tissue utilization of fatty acids and transfer of cholesterol from these lipoproteins to HDL-C (see Fig. 1).

## Effect of thyroid hormone on lipid metabolism

Thyroid hormone influences all aspects of lipid metabolism including synthesis, mobilization, and degradation. Thyroid hormone increases the activity of 3-hydroxy-3-methyl-glutaryl coenzyme A reductase, cholesteryl ester transfer protein (CETP), hepatic lipase, and lecithin:cholesterol acyltransferase [1, 2]. It also increases the expression of LDL receptors and HDL receptor scavenger receptor class-B, type I (see Fig. 1). Insufficient thyroid hormone levels would be predicted to be associated with increased serum total cholesterol (TC), LDL-C, and triglycerides (TG), accompanied by a decrease in HDL-C.

## Lipid changes with TSH values within the normal range

### Cross-sectional studies

The profound effect of thyroid status on lipid metabolism can be illustrated by several cross-sectional studies showing that as TSH increases even within the reference range, serum TC, LDL-C, and TG all increase, and HDL-C decreases [3–5]. For example, in one study of 3664 euthyroid individuals there was a significant association with increasing serum TSH levels and higher values for log TC and log TG. Interestingly, the effect of TSH seemed to be comprised of both a direct effect, independent of thyroid hormone levels, and an indirect effect exerted through thyroid hormone levels [5]. In another study of 30,656 individuals, as TSH increased within the normal range there were significant linear increases in TC, LDL-C, and TG, along with a significant linear decrease in HDL-C [3]. For men below 50 years, only the relationship between TG and TSH was maintained, whereas for women below 50 years the relationship was maintained for all lipids except HDL-C. When examining the relationship between TSH and lipids in the population older than 65 years, increasing TSH values were associated with worsening lipid profile, except for the group who were older than 80 years. Other studies extend these findings by showing associations between higher normal TSH values and metabolic syndrome [6–9] and worse cardiovascular outcomes [6], thus raising further concern for a higher risk of cardiovascular disease.

### Longitudinal studies

The data from longitudinal studies are less definitive than those from cross-sectional studies. In a study with baseline assessment of TSH levels and lipid profile, higher baseline TSH values were associated with higher TC, LDL-C, and log TG. At the 11-year follow-up, higher baseline TSH was only associated with higher TC and LDL-C in women, but not in men [10]. In another longitudinal study [11], higher TSH levels at baseline were associated with higher non-HDL cholesterol (difference between TC and HDL-C) and TG levels in men and lower HDL-C levels in both women and men at the 11-year follow up. Thus, compared with the previously mentioned data from their baseline study [3], the associations were not consistent between the sexes, and were also relatively modest. However, among individuals who remained free of thyroid disease, changes in TSH levels during follow-up were associated with accompanying changes in non-HDL cholesterol and triglyceride levels, with similar results being observed for women and men. Specifically, lipid levels increased among individuals with an increase in TSH levels and decreased among those with a decrease in TSH levels compared with people with no change in TSH levels [11]. The mean increase in non-HDL-C for a 1 mIU/L increase in TSH was 0.072 mmol/L in women and 0.102 mmol/L in men after adjustment for body weight and body mass index. For TG values, the mean increase in TG levels was 0.05 mmol/L in women and 0.108 mmol/L for men, each for a 1 mIU/L increase in serum TSH and after adjustment for body weight and body mass index.

## Lipid changes with subclinical hypothyroidism

In a recent study using both NHANES data and the Optum administrative claims database, 1.4% (3,500,678) of survey participants in the United States had untreated subclinical hypothyroidism [12]. Although not found consistently across all studies, the following lipid abnormalities have been documented in many studies of individuals with subclinical hypothyroidism: elevated TC, elevated LDL-C, increased TG, and decreased HDL-C [13]. The findings regarding TG and HDL-C are more varied, compared with the more consistent findings for TC and LDL-C. Increased coronary heart disease events and mortality from coronary heart disease have also been reported.

### Meta-analyses

The discrepancies in the findings regarding the effects of subclinical hypothyroidism on the components of the lipid profile seen in various studies has been attributed to the heterogeneity of the populations that have been studied [13, 14]. For example, in one recent study, using an initial control group of 10,827 individuals for a group of 685 participants with subclinical hypothyroidism, there were statistically significant increases in TC and LDL-C and a statistically significant decrease in HDL-C between the control group and groups with mild and more severe subclinical hypothyroidism. However, when a more rigorous control group was identifying by matching for age and sex, differences in the lipid profile were no longer found [15]. On the other hand, a meta-analysis using 35 studies, most of which were case-control studies, found that TC, LDL-C, and TG concentrations were significantly higher in individuals with subclinical hypothyroidism compared with euthyroid controls [16]. HDL-C levels were significantly lower [16].

A recent meta-analysis of studies on the lipid profiles of individuals with subclinical hypothyroidism accounted for population heterogeneity by including only cross-sectional studies in which those with subclinical hypothyroidism and control euthyroid participants were matched by age, sex, and body mass index. They included studies conducted before December, 2021 and identified 25 studies which contributed 3347 participants (1669 with subclinical hypothyroidism and 1688 with euthyroidism) [14]. This analysis showed that compared with the euthyroid group, individuals with subclinical hypothyroidism had significantly higher TC, LDL-C, and TG levels. They also had significantly lower HDL-C levels. Two additional study findings were that the LDL-C was the component of the lipid profile that was most affected by subclinical hypothyroidism and that the lipid profile changes were more pronounced in individuals with TSH values greater than 10mIU/L. This finding extends the conclusion of a previous metaanalysis of 41,931 participants (4526 with subclinical hypothyroidism and 37,405 with euthyroidism) from 16 observational studies which was published in 2014 [17]. This prior analysis found an increase in TC, LDL-C and TG in those with subclinical hypothyroidism, but did not find any change in HDL-C between the groups with subclinical hypothyroidism and euthyroidism.

### Degree of impact on components of the lipid profile

Combining the data from the various studies included in the recent meta-analysis suggests a differential effect of subclinical hypothyroidism on the different components of the lipid profile [14]. Generally, effects on the LDL-C were large size effects, whereas effects on TC, TG, and HDL-C were medium size effects. Confirming that an increase in LDL-C is the most robust finding associated with subclinical hypothyroidism include studies published since this meta-analysis. For example, in a study conducted in China, subclinical hypothyroidism was found to be associated with significant increases in LDL-C and TG [18]. The larger impact of subclinical hypothyroidism on LDL-C supports a role of subclinical hypothyroidism in the development of coronary heart disease. Increased LDL-C in patients with subclinical and overt hypothyroidism may enhance the formation of oxidized LDL [19], which may in turn play a role in the development of atherosclerosis and coronary heart disease [20].

### Subgroup analyses

Some studies have also conducted various subgroup analyses of effects on the lipid profile, including differences based on study location, degree of TSH elevation, age, sex, and body mass index. Examining the impact of degree of TSH elevation on lipid alterations in the recent meta-analysis [14], it was found that elevations in LDL-C, TC, and TG were greater in subgroup of patients with subclinical hypothyroidism and with TSH values greater than 10 mIU/L than in those with TSH values less than 10 mIU/L. However, this did not apply to the degree of decrease in HDL-C values. The greater impact of higher TSH values with respect to producing a more adverse lipid profile is in keeping with the finding that there is an increased risk of coronary heart disease events and mortality in those with greater TSH elevations, especially greater than 10 mIU/L [21]. The same meta-analysis [14] found greater differences in the TC, LDL-C and HDL-C compared with the euthyroid group in patients with subclinical hypothyroidism when comparing those aged between 21 and 55 years of age. Regarding sex, the increases in LDL-C and decreases in HDL-C were seen regardless of the percentage of women in the group being studied.

In another study, subgroup analyses showed that subclinical hypothyroidism was associated with significant elevation in TG and significant decrease in HDL-C in premenopausal women [18]. Other lipid changes were not significant in either pre-menopausal or post-menopausal women. Another analysis, using data from the REACTION study [22], examined the effect of participant age on the lipid elevation for various TSH elevations [23]. For example, in 60–69 years old participants, mild and significant subclinical hypothyroidism increased the prevalence of high TC approximately 1.50- and 2.27-fold, respectively, when compared with participants aged 40–49 years. Similarly, in the 60–69 years age group, mild and significant subclinical hypothyroidism increased the prevalence of high LDL-C approximately 1.26- and 1.88-fold, respectively, when compared with the 40–49 years old age group [23].

### Lipotoxicity and subclinical hypothyroidism

Lipid abnormalities have long been studied as a consequence of hypothyroidism. Recently there has been interest in whether the reverse could also be true and whether high lipid levels could also have an adverse effect on the function of the thyroid via a lipotoxicity mechanism. A case-control study was conducted in China in which 5033 individuals with subclinical hypothyroidism were matched with control individuals based on age, sex, and region using data from the REACTION study [22, 24]. The association between serum TG levels and the risk for subclinical hypothyroidism was then examined [24]. Elevated TG levels were found to be associated with increased risk of subclinical hypothyroidism in both men and women. Furthermore, the risk for subclinical hypothyroidism increased with progressive increases in the TG concentrations [24].

A recent study also took this different perspective and examined the relationship between the longitudinal lipid profile and progression of subclinical hypothyroidism using data from the REACTION study [22, 25]. Patients with subclinical hypothyroidism had documentation of their lipid profile on two occasions three years apart and the lipid changes were classified as a greater than 25% increase, versus a minor change, versus a greater than 25% decrease. The natural history of the subclinical hypothyroidism was also documented. The investigators used logistic regression models to assess the association between the changes in the lipid parameters and the natural history of subclinical hypothyroidism, including age, sex, body mass index, TSH, free triiodothyronine, free thyroxine, thyroid peroxidase antibodies, creatinine, smoking status, and alcohol consumption in the model. The investigators found that increase in TC levels increased the risk of progression to overt hypothyroidism, while a decrease in TC and TG levels was associated with a higher likelihood of regression to euthyroidism [25]. These findings were seen in both a retrospective cohort and a validation cohort. The findings raise the possibility that changes in TC and TG levels may also be prognostic factors that affect the likelihood that subclinical hypothyroidism will progress or resolve.

## Lipid changes with overt hypothyroidism

The previously mentioned study that used both NHANES data and the Optum administrative claims database, found the prevalence of hypothyroidism in the United States to be 9.6%, [12] compared with the prior estimate of 4.6% [26].

### Chronic hypothyroidism

Overt hypothyroidism would rarely be monitored without correction with thyroid hormone, so most data regarding lipid abnormalities stems from studies of subclinical hypothyroidism. Overt hypothyroidism generally retains all the lipid abnormalities characteristic of subclinical hypothyroidism (see Table 1), with the greater derangement in the lipid parameters being associated with the degree of TSH elevation [13, 14, 17, 27–30].

**Table 1:** Potential effects of thyroid status and thyroid hormone treatment on lipid parameters

Thyroid status	Total cholesterol	LDL cholesterol	HDL cholesterol	Triglycerides
TSH within upper part of normal range (compared with lower part of normal range)	Increased	Increased	Decreased	Increased
Subclinical hypothyroidism (compared with euthyroid)	Increased	Increased	Decreased or normal	Increased or normal
Treated subclinical hypothyroidism (compared with untreated)	Decreased	Decreased	Unchanged	Decreased
Overt hypothyroidism (*spontaneous, longer duration*) (compared with euthyroid)	Increased	Increased	Decreased or normal	Increased or normal
Treated overt hypothyroidism (compared with untreated)	Decreased	Decreased	Decreased	Decreased
Overt hypothyroidism (*of short duration)* (compared with previously euthyroid or treated)	Increased	Increased	Increased	Increased or normal

The following studies provide some examples of the changes in lipid parameters seen with overt hypothyroidism [28, 29, 31–33]. In one study of 58 individuals presenting with overt hypothyroidism and a mean TSH of 42 mIU/L, 34.5% had high TC levels, 56.9% had high LDL-C levels, 69% had high TG levels, and 62.1% had low HDL-C levels [33]. Another study examined 268 patients presenting with primary hypothyroidism (mean TSH not provided) who were not receiving lipid-lowering therapy. Their median lipid levels were as follows: TC 257 mg/dL, LDL-C 180 mg/dL, TG 125 mg/dL, and HDL-C 50 mg/dL [28]. In a third study 23 patients with overt hypothyroidism were documented as having the following mean values: TC 240 mg/dL, LDL-C 154 mg/dL, TG 170 mg/dL, and HDL-C 53 mg/dL. The values for euthyroid controls were TC 198 mg/dL, LDL-C 124 mg/dL, TG 115 mg/dL, and HDL-C 50 mg/dL [31]. In a group of 24 individuals with hypothyroidism and a mean TSH of 43.6 mIU/L, mean lipid values were TC 322 mg/dl, LDL-C 232 mg/dL, TG 148 mg/dL, and HDL 54 mg/dL [32]. Twenty three hypothyroid women had a mean TSH level of greater than 75 mIU/L which was associated with a mean LDL-C of 182.5 mg/dL and a mean HDL-C of 62.3 mg/dL [29]. In a small group of 17 patients with severe hypothyroidism characterized by a mean TSH of 91.4 mIU/L, lipid levels were as follows: TC 271 mg/dL, LDL-C 198 mg/dL, TG 133 mg/dL, and HDL 50 mg/dL [34]. Most recently, 41 individuals with hypothyroidism and a mean TSH of 50.6 mIU/L were found to have elevated TC and LDL-C compared with a control group with a TSH of 2.06 mIU/L. The values were a TC of 187 mg/dL (compared with 146 mg/dl in the control group) and a LDL-C of 124 mg/dL (compared with 93 mg/dL in the control group) [35].

### Acute onset of hypothyroidism

There are also some studies that serve to illustrate the effects of hypothyroidism of relatively short duration on the lipid profile. In one study 60 patients underwent thyroidectomy for an unspecified reason, were not started on thyroid hormone, and had lipid profiles measured 3 weeks later [36]. Compared with a group of control individuals, those with post-surgical hypothyroidism (serum TSH of approximately 70 mIU/L) had higher TC, LDL-C, TG, and TC/HDL-C ratio [36]. There was no significant change in HDL-C. Three other studies examined lipid profiles in thyroidectomized patients who were hypothyroid while awaiting radioactive iodine treatment [37–39]. In one study of 27 patients with a mean TSH concentration of 91 mIU/L [37] there were alteration in all lipid parameters: increased TC, LDL-C, TG, and HDL-C. Cholesterol efflux, which was considered to be a measure of HDL function was decreased [37]. A second study of 75 patients with a mean TSH value of 62 mIU/L [38] also found significant elevation in TC, LDL-C, TG, and HDL-C. The final study included 18 women with a mean TSH concentration of 72 mIU/L. TC, LDL-C, and HDL-C were all increased, whereas there was no significant change in TG. Although HDL-C increased, there was no increased capacity of the HDL-C to receive lipids or the activity of paraoxonase-1 the anti-oxidation enzyme associated with HDL. Two of these studies may provide data suggesting that although HDL-C concentrations may increase in this model with onset of hypothyroidism over a several week period, the HDL-C may be relatively dysfunctional [37, 39].

## Treatment of hypothyroidism and the lipid profile

Treatment of hypothyroidism is associated with improvement in abnormal lipid parameters [40, 41]. A recent meta-analysis is helpful in illustrating the changes that occur in the lipid profile in both subclinical and overt hypothyroidism [40]. For overt hypothyroidism, the meta-analysis showed that initiation of levothyroxine was associated with a decrease in TC of 58.4 mg/dL, a decrease in LDL-C of 41.2 mg/dL, a decrease in TG of 27.3 mg/dL, and a decrease in HDL-C of 4.2 mg/dL, with all changes being significant. The magnitude of the change in the lipid parameters was similar for studies with less than 3 months of follow up compared with those with more than 6 months of follow up. In the case of subclinical hypothyroidism 82% of studies examined treatment in patients with TSH values of 5–10 mIU/L, whereas 18% examined treatment in patients with TSH values of greater than 10 mIU/L. Levothyroxine treatment was associated with a significant decrease in TC of 12 mg/dL, a significant decrease in LDL-C of 11 mg/dL, and a significant decrease in TG of 4.5 mg/dL. There was no significant change in HDL-C.

One of the studies included in the meta-analysis discussed above was a retrospective study of 70 patients with overt hypothyroidism [28]. Levothyroxine treatment reduced their mean TSH from 62 to 1.2 mIU/L. Accompanying this normalization of TSH was a significant decline in median TC (264 mg/dL reduced to 222 mg/dL), LDL-C (187 mg/dL reduced to 152 mg/dL), TG (114 mg/dL reduced to 100 mg/dL) and HDL (54 mg/dL reduced to 50 mg/dL). One of the studies of treating subclinical hypothyroidism included in the meta-analysis discussed above was a randomized open label trial [42]. The intervention group received levothyroxine which lowered serum TSH from 5.9 to 2.8 mIU/L. Accompanying treatment, TC decreased from 225 to 209 mg/dL, LDL-C decreased from 129 to 125 mg/d, and HDL-C decreased from 54 to 52 mg/dL. The change in TG was no different between the intervention and control groups. The study also suggested that patients benefited from levothyroxine treatment regardless of their baseline TSH or TC concentrations [42].

## Metabolomics, lipidomics

Recently a metabolomic study was conducted in 126 individuals, including 45 with overt hypothyroidism, 41 with subclinical hypothyroidism, and 40 euthyroid controls [35]. The plasma metabolomic patterns in the two hypothyroid groups were significantly different from those of the control group. On the other hand, metabolite alterations between the two hypothyroid groups were notably similar. Pathway enrichment analysis found that hypothyroidism significantly impacted bile acid biosynthesis, steroid hormone biosynthesis, lysine degradation, tryptophan metabolism, and purine metabolism. Sixty-five metabolites were found to be significantly associated with levels of TSH, free thyroxine, thyroid peroxidase antibody, or thyroglobulin antibody. The authors were able to validate 17 metabolic biomarkers which successfully distinguished the 3 groups from each other [35]. Serum lipidomic analyses have also been performed to help identify the serum biomarkers and metabolic pathways associated with hypothyroidism. A recent study enrolled 175 individuals for a discovery set and 300 for a validation set (TSH values 23–27 mIU/L) [43]. Using this approach 6 potential biomarkers were identified for hypothyroidism which were involved in glycerophospholipid metabolism. A biomarker panel using the six hypothyroidism biomarkers showed good ability to distinguish patients with hypothyroidism from the healthy population with an area under the curve of 0.97, sensitivity of 86%, and specificity of 96% [43]. Similar approaches have been taken using proteomics and metabolomics to identify a combination of proteins and metabolites that can distinguish hypothyroidism from the euthyroid state [44]. Such studies should enhance our understanding of the pathologic changes in metabolism occurring in patients with hypothyroidism.

## Conclusion

Thyroid hormone affects many aspects of lipid metabolism. Both subclinical and overt hypothyroidism are associated with derangements in the lipid profile. The degree of derangement is generally proportional to the degree of TSH elevation, even to the extent that lipid abnormalities can be seen with TSH values in the upper part of the normal range. Cross-sectional studies suggest that an increase in LDL-C is the most robust finding associated with subclinical and overt hypothyroidism, with evidence for increased risk of adverse metabolic and cardiovascular impact. Thyroid hormone treatment reverses the lipid abnormalities. The expectation that treatment of hypothyroidism would be associated with reduced risk of metabolic and cardiovascular consequences merits the consideration of hypothyroidism as a non-communicable disease with significant adverse impact on population health. This would pave the way for studies testing this hypothesis to be conducted.
